# Errata

**Published:** 1871-08

**Authors:** 


***Errata.***

Page 341, in prescription given, read gr. $\frac{1}{3}$ of the strychnia, instead of $\frac{1}{73}$. Please correct with a pen.

In the valuable article on Hypodermic Medication, published last month, on page 421, for "Austin" read *Anstie.* In line 6, page 433, for "inserted" read *inverted.* Be sure and correct this last with a pen, as otherwise accidents might possibly ensue.

It is impossible always to avoid these typographical errors, although we flatter ourselves that the JOURNAL is as free from them as any other periodical extant.

